# Diabetes as a predictor of COVID-19 preventive behaviors

**DOI:** 10.3389/fpsyg.2025.1496183

**Published:** 2025-04-16

**Authors:** Ines Gonzalez Casanova, Rachel Klingensmith, Barbara A. Myers, Farrah Anwar, Mary de Groot

**Affiliations:** ^1^Indiana University Bloomington School of Public Health, Bloomington, IN, United States; ^2^Indiana University School of Medicine, Diabetes Translational Research Center, Indianapolis, IN, United States

**Keywords:** COVID-19, psychosocial, health beliefs model, theory of planned behavior, type 1 diabetes, type 2 diabetes

## Abstract

**Introduction:**

We explored if diabetes status predicted differences in behavioral pathways associated with staying home at the beginning of the coronavirus-19 infectious disease (COVID-19), wearing a mask, and vaccinating in a convenience sample of US adults over a 12-month period of the COVID-19 pandemic (from May 2020 through June 2021).

**Methods:**

We included participants who completed web-based surveys in May–June, 2020 (baseline), and at the 6-, 9-, 11- and 12- months follow-ups (*n* = 966). We collected information on demographic characteristics (baseline) and surveys with Likert-scale type questions to assess Health Beliefs Model and Theory of Planned Behavior constructs related to staying home (6-month), wearing masks in public spaces (9-month), and receiving the COVID-19 vaccine (11- and 12- month). Structural equation modeling was conducted to assess behavioral pathways and direct and indirect associations with diabetes.

**Results:**

Constructs of the Theory of Planned Behavior and the Health Beliefs Model explained intention to stay home, to wear a mask, to vaccinate, and COVID-19 vaccination status. Diabetes status predicted intention to stay home directly (*β* = 0.21, *p* < 0.05) and indirectly through perceived severity (β = 0.11, *p* < 0.01). Overall, diabetes status was not associated with intention to wear a mask or vaccination.

**Conclusion:**

Findings from this study highlight relevant pathways that can be leveraged to promote preventive behaviors in people with diabetes.

## Highlights

- *What is already known?* People with diabetes were particularly impacted at the beginning of the COVID-19 pandemic and faced unique psychosocial determinants. Diabetes may play a role predicting behavioral pathways associated with COVID preventive behaviors including staying home, wearing a mask, and receiving a vaccine.
- *What is added?* Diabetes predicted intention to stay at home but not intention to mask or engage in vaccination behaviors in this longitudinal study. A model combining Health Beliefs Model and Theory of Planned Behavior constructs was effective in explaining intention to stay home, wear a mask, and vaccinate in a US sample of people with and without diabetes.
- *What are the implications for public health practice?* This information can be used to design preventive interventions for people with and without diabetes.

## Introduction

The pandemic of coronavirus infectious disease 2019 (COVID-19) resulted in over 765 million cases and more than 6.9 million deaths worldwide, with over a million of those deaths occurring in the US (Johns Hopkins University, 2023). Individual preventive behaviors such as staying home, especially at the beginning of the pandemic, wearing masks in public spaces, and getting the COVID-19 vaccine when it became available, were essential to control the spread of the virus and minimize morbidity and mortality (Hamimes et al., 2023; Mahmoudi and Xiong, 2022). To learn from the COVID-19 pandemic and improve future public health interventions, it is important to understand the behavioral pathways associated with these COVID-19 preventive behaviors.

Diabetes status may have been associated with these preventive behaviors during the COVID-19 pandemic. People with diabetes were particularly impacted by the COVID-19 pandemic. Diabetes is associated with worse disease symptoms, complications, and mortality from COVID-19 (Gregory et al., 2021). Moreover, people with diabetes faced different barriers during the COVID-19 pandemic because they were affected by concerns about medications shortages and costs as well by difficulties accessing care (Fisher et al., 2020). Similarly, there were concerns about potential adverse events of the COVID-19 vaccine that specifically affected people with diabetes. These may have impacted the behavioral pathways related to staying at home, wearing a mask, and getting a vaccine in people with diabetes.

Individual behavioral models have long been applied to understand health-related behaviors in the context of infectious disease prevention. The Health Belief Model (HBM) and the Theory of Planned Behavior (TPB) are two widely used models that have been applied to understand and develop interventions to prevent infectious diseases (Carpenter, 2010; Kretzer and Larson, 1998). The HBM was specifically developed to understand behavioral pathways related to vaccine acceptance. It states that people’s beliefs influence their health-related behaviors and that individuals will likely act when they perceive a risk or threat but only if the perceived benefits outweigh the barriers (Janz and Becker, 1984; Rosenstock, 1974). The TPB is based on the premise that individuals make logical planned decisions to engage in specific behaviors based on the information available to them (Ajzen, 1991). Hence, according to this theory, a large proportion of variations in behavior can be explained through intentions, which in turn are determined by attitudes towards the behavior, subjective norms, and perceived behavioral control. More recently, Callow et al. (2020) developed a model combining constructs from the Health Belief Model and the Theory of Planned Behavior to predict decisions about staying home among older adults during the COVID-19 pandemic.

In a large observational study including over 2000 US adults, we found that participants with type 2 diabetes had more depression, lower resilience and more COVID-19 risk factors and medical comorbidities compared with participants without diabetes (Myers et al., 2022). The purpose of this follow-up study was twofold. First, we aimed to assess the effectiveness of the model combining HBM and TPB explaining staying at home, mask wearing, and vaccination behaviors. Secondly, weexplored direct and indirect associations of diabetes with these behaviors.

## Methods

### Study design

This was a cohort study where participants were recruited through a variety of methods (Myers et al., 2022) and were asked to complete a web-based survey in May–June, 2020 (baseline), November–December, 2020 (6- month follow-up), Feb-March, 2021 (9- month follow-up) and March–June 2021 (11- and 12-month follow-up). Surveys included basic demographic and social information, health beliefs related to COVID-19 and intention to stay home (6- month follow-up), intention to wear a mask (9 month follow up), intention to receive the COVID-19 vaccine (11- month follow up, the point at which vaccines were widely available to the general public) and COVID-19 vaccination (12 month follow-up). Participants also completed monthly surveys with additional information, including a survey between March and April 2021 where they provided information about health beliefs related to vaccination. The study was approved by the Indiana University IRB.

### Recruitment and procedures

Participants were recruited through the NIH’s Research Match website, the Indiana CTSI’s All IN for Health registry, and through social media and other postings by Diabetes Sisters, Inc., an online support group for people with diabetes and their families. Detailed recruitment information can be found in a previous publication describing the baseline results for this study (Myers et al., 2022). Participants were provided with a link to the study’s online consent form which then proceeded to the survey. Participants were sent a link to the survey monthly. At baseline and months 6 and 12, a gift card raffle was held for survey respondents. For every 100 participants at each respective time point, one study ID was selected at random to receive a $50 electronic gift card.

### Measures

Participants were asked to complete most study measures at baseline, and 6-, 9-, 11-and 12- month follow-ups. To reduce participant burden, each data collection focused on a different preventive behavior (e.g., staying home, masking, vaccination). All measures are validated and widely utilized within the scientific literature as described below.

#### Demographics and medical history

Demographic and medical history information, including COVID history, was collected at baseline and month 12. Demographic information included sex, race, education, income, home ownership and financial wellbeing. Medical background included questions on diabetes status, comorbid conditions (heart disease, cancer, depression diagnosis, etc.). This information was used to calculate the number of COVID risk factors per participant as well as the number of comorbidities per participant. Risk factors included diagnoses of diabetes, heart disease, cancer, cardiovascular disease and asthma. We also asked questions regarding COVID diagnosis, employment status, and vaccine status (at month 12).

#### Behavioral predictors of COVID-19 prevention

Fourteen items designed to assess constructs from the Theory of Planned Behavior and the Health Beliefs Model related to intention to stay home, intention to mask, and intention to receive the COVID-19 vaccine included in the month 6-, 9- and 11- month data collection. Each item presented a 7-point Likert scale where higher scores represented greater agreement with the statements presented and lower scores greater disagreement. The items related to constructs in the HBM and TPB as follows:

- Benefits (HBM): 1. [behavior] will keep me safe from COVID-19, 2. [behavior] will help diminish the spread of COVID-19. Definition: expected gains or positive consequences of the behavior.
- Barriers (HBM): 3. Other problems are more important than COVID-19, 4.[behavior] is too painful, 5.[behavior] is too difficult, 6. [behavior] is not good for physical health, 7. [behavior] is not good for mental health, 8 [behavior] will harm my finances. Definition: factors precluding or limiting the implementation of the behavior.
- Perceived Susceptibility (HBM): 9. It is likely that I will get COVID-19. Definition: participant’s belief about their likelihood of developing the disease.
- Perceived Severity (HBM): 10. If I get COVID-19, it will be serious. Definition: the negative consequences the participant associated with the event or outcome (in this case COVID-19)
- Attitudes (TPB): 11. [behavior] is a good idea, 12. I recommend [behavior]. Definition: a participant’s positive or negative evaluation of performing the behavior.
- Subjective norms (TPB): 13. Most people think that I should [behavior], 14. It is expected that I [behavior]. Definition: participant’s perception of social pressure to perform or not perform the behavior.
- Perceived behavioral control (TPB): 15. [behavior] is beyond my control. Definition: a participant’s belief in their ability to perform a specific behavior.

### Statistical analyses

For these analyses, we included participants with information at baseline, 6, 9, 11 and 12 months. Analyses for this data set included means and frequencies for all variables. Chi squares were performed to assess different distributions between groups on categorical variables. For continuous variables, ANCOVAs were conducted using standard covariates of age, race, ethnicity, income, and, for repeated measures.

For the structural equations modeling, we conducted path analysis, a unique case of structural equation model where variables are assumed to be measured without error (Jeon, 2015). The first aim of this analysis was to explore associations of behavioral constructs from the HBM and the TPB with intention to stay home, intention to wear a mask, and receiving the COVID-19 vaccine by the 12-months follow-up of the study as an outcome and compare the combined model with individual models including only HBM or only TPB constructs. The second aim was to assess direct and indirect pathways through behavioral constructs of diabetes status predicting COVID-19 vaccination at the 12-month follow-up.

Models were built based on theory and evaluated using the root mean square error of approximation (RMSEA), Tucker–Lewis index (TLI), and the Standardized Root Mean Square Residual (SRMR), which has been recommended as the best indicator of model fit for ordinal outcomes (Shi and Maydeu-Olivares, 2020). Models with RMSEA <0.10 or SRMR <0.08 were not considered. All models were adjusted using baseline sociodemographic variables. Nine theoretically sound models with acceptable fit indicators were used for the analysis (one per each model per each preventive behavior). Path analysis was conducted using Lavaan in R 4.1.2 (Rosseel, 2012) with ULSMV, which has been recommended to estimate fit indexes in models with ordinal outcomes.

## Results

### Demographics

A total of 966 participants with information at baseline, 6, 9, 11, and 12 months were included in this study. At baseline, participants were on average 53 (SD = 16.4) years old, majority female, and highly educated. Participants with type 1 or type 2 diabetes (*n* = 168) were older, a greater proportion were male and less likely to have annual incomes over $100,000 USD compared to participants without diabetes (Table 1). Overall, 92% (888) of participants had received at least one COVID-19 vaccine by the 12 months follow-up.

**Table 1:** Baseline demographic characteristics of the analytical sample (*n* = 966) by diabetes status.

	Participants without diabetes (*n* = 798) % (*N*)	Participants with diabetes (*n* = 168) % (*N*)	*p*-value
Age (years; *M* ± S.D.)	52.4 ± 16.6	58.1 ± 14.4	<0.001
Sex			
Female	82.2 (650)	70.8 (119)	<0.001
Male	18.6 (154)	29.2 (49)	
Race			
White	91.2 (728)	91.7 (154)	0.278
Black	2.9 (23)	3.6 (6)	
Other	4.9 (39)	4.2 (7)	
Hispanic or Latino	4.0 (32)	2.4 (4)	0.311
Annual income (USD)			
$ 0–10,000	2.5 (20)	3.0 (5)	0.018
$10,001-20,000	4.1 (33)	7.1 (12)	
$20,001-40,000	14.3 (114)	21.1 (37)	
$40,001-60,000	15.7 (125)	16.1 (27)	
$60,001 – 80,000	16.4 (131)	14.3 (24)	
$80,001-100,000	13.0 (104)	15.5 (26)	
>$100, 000	34.0 (271)	22.0 (37)	

*p-values calculated using ANOVAS and Chi-square tests.*

### Unadjusted associations between diabetes status and behavioral predictors of COVID-19 prevention

Intention to stay home during the 6-month follow-up among study was higher among participants with diabetes (including type 1 and 2 diabetes) compared to participants without diabetes (Table 2). Perceived susceptibility and perceived severity were also higher among participants with diabetes at this follow up. Overall, study participants had high intentions to wear a mask in public (6.7 ± 0.0) and to get the COVID-19 vaccine (6.4 ± 0.1) at the 9- and 11- month follow-up, respectively, with no differences by diabetes status. Perceived severity remained consistently higher among participants with diabetes compared to those without diabetes throughout the study. Subjective norms related to vaccination were higher among people with diabetes (4.0 ± 0.0) compared to people without diabetes (3.9 ± 0.0) (Table 2).

**Table 2:** Behavioral predictors of staying at home, wearing a mask, and vaccination by diabetes status (*n* = 966).

	People without diabetes (*N* = 798) *M* ± S.D.	People with diabetes (*N* = 168) *M* ± S.D.	*p*-value
Staying home November–December 2020			
Intention	5.3 ± 1.8	5.7 ± 1.6	0.007
Benefits	5.9 ± 1.3	5.8 ± 1.1	0.460
Barriers	3.0 ± 1.3	2.9 ± 1.3	0.998
Perceived susceptibility	3.4 ± 1.5	3.1 ± 1.4	0.001
Perceived severity	4.8 ± 1.8	5.8 ± 1.4	<0.001
Attitudes	5.9 ± 1.5	5.9 ± 1.3	0.992
Subjective norms	4.9 ± 1.7	5.3 ± 1.4	0.083
Perceived behavioral control	2.8 ± 1.9	2.7 ± 1.8	0.439
Wearing masks in public February–March 2021			
Intention	6.7 ± 0.9	6.8 ± 0.6	0.085
Benefits	6.0 ± 1.3	6.1 ± 1.1	0.116
Barriers	1.7 ± 1.1	1.7 ± 1.0	0.998
Perceived susceptibility	2.6 ± 1.3	2.6 ± 1.4	0.704
Perceived severity	4.6 ± 1.8	5.7 ± 1.3	<0.001
Attitudes	6.4 ± 1.3	6.5 ± 1.0	0.158
Subjective norms	3.9 ± 0.5	4.0 ± 0.5	0.088
Perceived behavioral control	6.5 ± 1.0	6.5 ± 0.9	0.477
Receiving the COVID-19 vaccine April–May 2021			
Intention	6.5 ± 1.5	6.5 ± 1.2	0.687
Benefits	6.1 ± 1.2	5.9 ± 1.3	0.203
Barriers	1.6 ± 0.8	1.7 ± 0.9	0.316
Perceived susceptibility	2.2 ± 1.3	2.4 ± 1.6	0.06
Perceived severity	4.6 ± 1.9	5.3 ± 1.8	<0.001
Attitudes	6.4 ± 1.3	6.5 ± 1.1	0.787
Subjective norms	3.9 ± 0.5	4.0 ± 0.5	0.012
Perceived behavioral control	5.9 ± 1.5	5.8 ± 1.5	0.262

Scores reflect individual or composite answers to a 7-point scale. Values are mean ± standard deviation. *p*-values calculated using ANOVAS.

### Comparison of models including only HBM constructs, only TPB, and a combination of both

We identified nine models – three for each behavior – that met the inclusion criteria of having a RMSEA <0.10 and a SRMR <0.08. Models including the Theory of Planned Behavior constructs explained a greater proportion of the variability in intention to stay home (*R*^2^ = 0.55–0.58) and intention to wear a mask (*R*^2^ = 0.62–0.63), compared to the models including only HBM constructs (*R*^2^ = 0.44 for both intention to stay home and intention to wear a mask). The combined (HRM + TPB) model explained the greatest proportion of the variability for vaccination (*R*^2^ = 0.68) compared to HBM (*R*^2^ = 0.60) and TPB (*R*^2^ = 0.55) (Table 3).

**Table 3:** Path analysis coefficients and fit indicators for models including health belief model constructs, theory of planned behavior constructs, or a combination of constructs form both models (*N* = 966 participants).

Outcome	Intention to stay home (month 6)			Intention to wear mask (month 9)			Vaccinated (month 11–12)		
*N* = 966/ Model #	HBM 1	TPB 2	Combo 3	HBM 4	TPB 5	Combo 6	HBM 7	TPB 8	Combo 9
Intention								0.55**	0.50**
Attitudes		0.57**	0.41**		0.37**	0.31**		0.88**	0.60**
Barriers			−0.34**			−0.47**			−0.64**
Benefits			0.54**			0.48**			0.43**
Diabetes		0.01	0.09		0.12				
Subjective norms		0.65**	0.44**		0.14*	0.15*		0.23**	0.13*
Diabetes								0.28	
Per. Beh. Control		−0.02	0.04		0.52**	0.34**		0.46**	0.16**
Barriers									−0.38**
Benefits									0.12**
Diabetes								−0.01	0.16*
Per. Susceptibility	0.04		0.06	0.06**		0.06**	−0.08		−0.10*
Diabetes	−0.20*			0.05			0.18		0.21**
Perceived Severity	0.21**		0.14**	0.07**		0.04**	0.13*		0.12*
Diabetes	0.85**		0.80**	0.89**		0.84**	0.65**		0.63**
Barriers	−0.55**		−0.28**	−0.24**		−0.07	−0.80**		−0.31**
Benefits	0.47**		0.07	0.27**		0.04	0.42**		0.23**
Diabetes	0.32**	0.28**	0.21*	0.03	0.06	0.02	0.40	0.18	0.30
*R* ^2^									
Vaccinated							0.60	0.55	0.68
Intention	0.44	0.55	0.58	0.44	0.62	0.63		0.71	0.71
Attitudes		0.00	0.49		0.00	0.64			0.65
Per. Susceptibility	0.00			0.00			0.00		0.00
Perceived Severity	0.05		0.04	0.04		0.04	0.03		0.02
Model Fit (scaled)									
Tucker-Lewis Index	0.99	0.96	0.94	0.95	0.86	0.95	0.92	0.99	0.97
RMSEA	0.02	0.07	0.04	0.03	0.08	0.03	0.03	0.03	0.02
SRMR	0.03	0.00	0.04	0.06	0.00	0.05	0.02	0.02	0.03

All models were adjusted by race, Hispanic or Latino Ethnicity, income, age, and sex as direct effects to the outcome. Models with an RMSEA > 0.08 or SRMR >0.06 were not considered. Vaccination was a binary outcome and intention was an ordinal variable with 7 categories. Analyses were conducted using ULSMV estimator in R Lavaan 0.6–12. The table shows the following pathways, whereas more exogenous variables are denoted by lighter shading: Models 1,4 – intention ~ benefits+ barriers+ perceived_susceptibility+ perceived_severity + diabetes; perceived_susceptibility ~ diabetes; perceived severity ~ diabetes. Model 2,5 – intention ~ attitude+ subjective_norms+ perceived_behavioral_control+ benefits+ barriers+ diabetes; attitude ~ db; Models 3,6 - intention ~attitude+subjective_norms+ perceived_behavioral_control + benefits + barriers+ perceived_susceptibility+ perceived_severity; attitude ~ benefits+ barriers+ diabetes; perceived severity ~ diabetes; Model 7- vaccination ~ benefits+ barriers+ perceived_susceptibility+ perceived_severity +diabetes; perceived_susceptibility ~ diabetes; perceived severity ~ diabetes. Model 8- vaccination ~ intention +diabetes; intention ~ attitude+ subjective_norms+ perceived_behavioral_control+ benefits+barriers+diabetes. Model 9- vaccination ~ intention + benefits+ barriers+ perceived_susceptibility+ perceived_severity + diabetes; attitude ~ benefits+ barriers; perceived_susceptibility ~ diabetes; perceived severity ~ diabetes.*p < 0.05, **p < 0.01.

### Associations of diabetes status with pathways related to COVID-19 preventive behaviors

**Figure 1:**
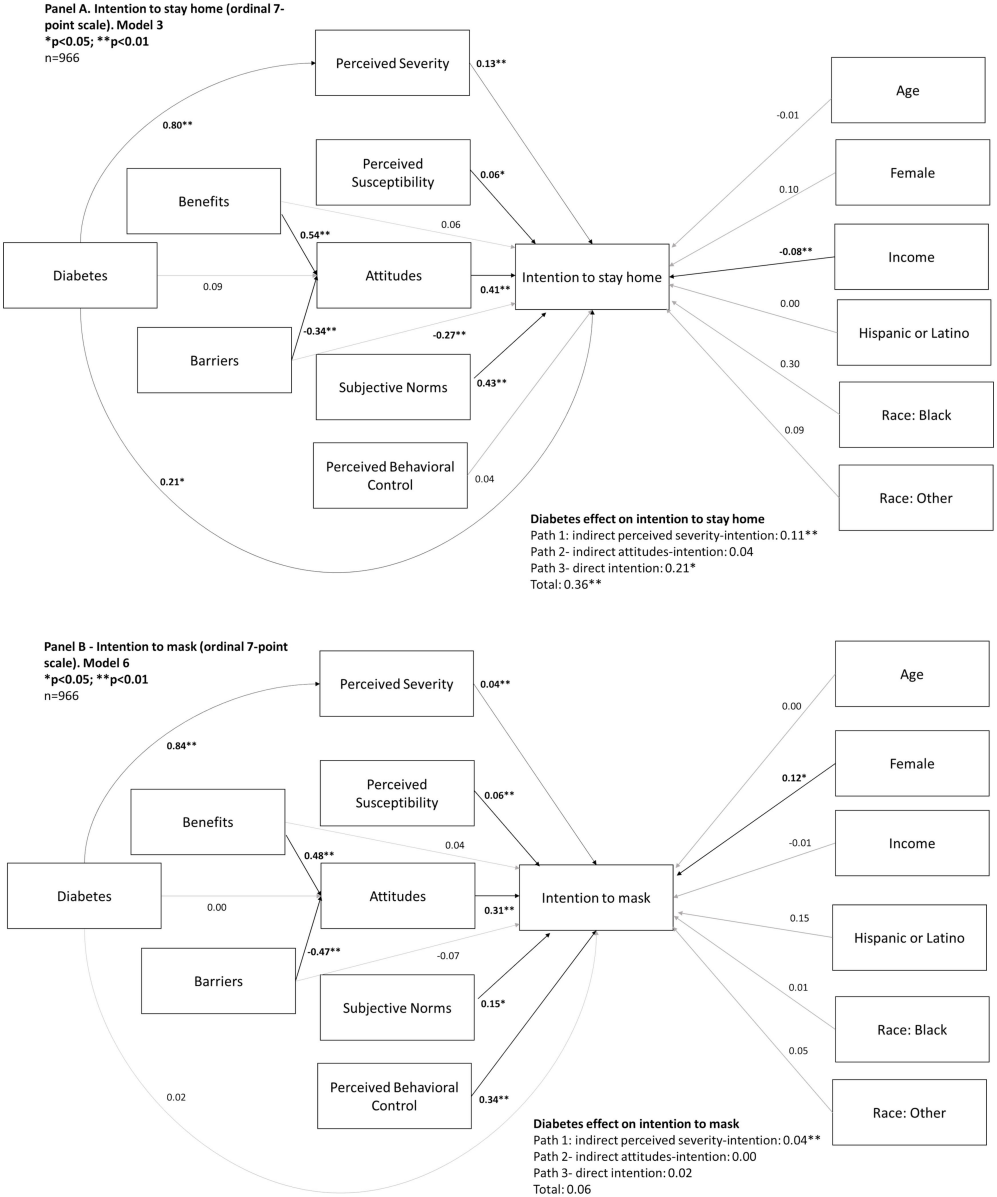
Behavioral pathways predicting intention to stay home (Panel **A**; November–December, 2020) and intention to mask (Panel **B**; February–March, 2021) during the COVID-19 pandemic in a sample of 966 U.S. adults.

**Figure 2:**
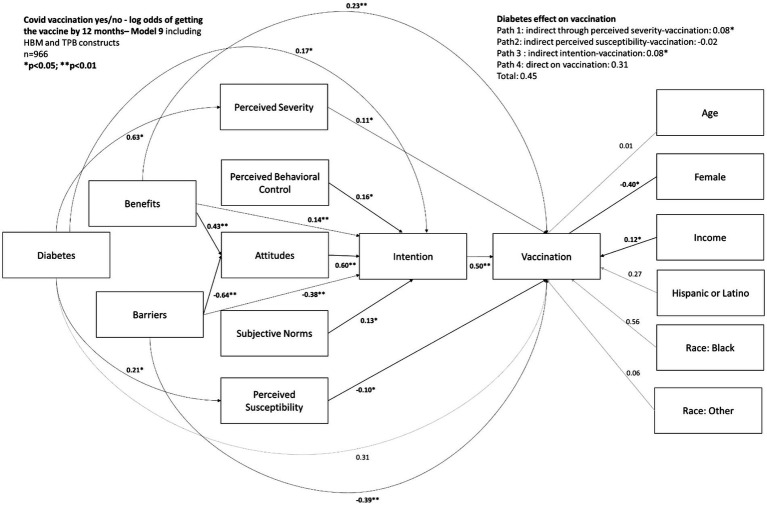
Behavioral pathways predicting COVID-19 vaccination by June 2021 in a sample of 966 U.S. adults.

Diabetes status was a significant predictor of intention to stay home (*β* = 0.36; *p* < 0.01) There was a significant direct effect (β = 0.21; *p* < 0.05) and an indirect effect mediated through perceived severity (β = 0.21; *p* < 0.01) (Figure 1A). Diabetes status was not a significant predictor of intention to wear a mask in public; there was a significant indirect effect mediated through perceived severity (β = 0.04; *p* < 0.01) but the total effect was not significant (Figure 1B). Overall, diabetes status did not significantly predict vaccination (*p* > 0.05), but there was a significant indirect effect through severity (β = 0.08; *p* < 0.05) and a significant indirect effect through intention (β = 0.08; *p* < 0.05) (Figure 2).

## Discussion

In this study, we report on longitudinal data collected from adults with and without diabetes at 6-, 9-, 11- and 12-months following baseline assessment near the start of the COVID-19 pandemic in the U.S. We tested behavioral constructs from two behavioral theories using a model that was theoretically sound and demonstrated good fit indicators. The strength of the overall model also allowed us to test mediation of behavioral constructs such as attitudes and intention in the association between diabetes and COVID-19 vaccination. A number of key findings emerged. First, the combined model (HBM + TPB) proved to be superior to each model individually, accounting for the greatest among of shared variance across all 3 infection prevention behaviors. These findings were consistent with the work by Callow et al. (2020) that was tested in a sample of older adults focused on social isolation. Our findings demonstrate that these relationships extend to a wider range of age and prevention behaviors.

We also observed that diabetes status was a significant predictor of perceived susceptibility and perceived severity of COVID-19 throughout the 12-month period of data collection. This finding is consistent with the public health messaging and early scientific findings that people with diabetes, and particularly those with obesity, had higher hospitalization and mortality rates than those without obesity and diabetes. Interestingly, diabetes status was a direct significant predictor of intention to socially isolate, but did not meet significance as a direct predictor of intention to wear masks later in the pandemic period. This may be attributed to relatively high levels of intention to wear masks in those with and without diabetes, making differences across the groups difficult to detect. It may also be attributable to study findings released later in the pandemic that suggested that pre-infection levels of hemoglobin A1c predicted worse morbidity and mortality outcomes in people with diabetes and COVID-19 (Ratzki-Leewing et al., 2022).

We also observed that higher income and sex were significant predictors of intention to stay home and mask, and/or COVID-19 vaccination behaviors. In general, these findings are consistent with prior data (Ben-Umeh and Kim, 2024; Myers et al., 2022; Papageorge et al., 2021; Tilchin et al., 2021). An exception is the inverse association between income and intention to stay home, which is not consistent with what had been reported before (Papageorge et al., 2021; Tilchin et al., 2021), and could be attributed to the overall high income of our study sample.

Finally, findings from the combined models demonstrated that perceived benefits of prevention behaviors and barriers to engaging in preventive behaviors were significant predictors of attitudes toward all three behaviors. These data demonstrate that using multiple model constructs allows us to better understand disease prevention behaviors previous studies had highlighted the importance of perceived barriers and attitudes explaining COVID-19 preventive behaviors (An et al., 2021; Anaki and Sergay, 2021). The success of this combined model including these constructs explaining a large proportion of the variability in intention to stay home, wear a mask, and COVID-19 vaccination in our study further supports the value of this integrated approach (An et al., 2021).

Limitations of the study include the use of a convenience sample of adults who had access to email and computers that enabled them to be recruited and engage in study surveys over time. This limits generalizability of these findings to the full spectrum of socioeconomic status and indicates a need to further evaluate elements of the HBM and TPB models in samples that represent a broader socioeconomic range. In addition, sample retention across the five time points used in this study decreased the sample size and has implications for the generalizability of findings. However, a large enough sample was retained to be able to conduct complex modeling without significant loss of statistical power to detect effects. A limitation of our modeling was the use of categorical and ordinal outcome variables as exposures that decreased the variability of the model and our ability to test further paths and associations.

In sum, this study found that a combination of the Health Beliefs Model and Theory of Planned Behavior constructs significantly predicted intention and use of prevention behaviors for COVID-19. People with diabetes demonstrated increased feelings of perceived severity and susceptibility to COVID-19 which, in turn, influenced attitudes toward prevention behaviors. These findings can be used to consider ways to engage at risk populations in prevention strategies in the context of future epidemics and pandemics. Future research can leverage these findings to develop interventions that promote preventive behaviors in people living with diabetes.

## Data Availability

The raw data supporting the conclusions of this article will be made available by the authors, without undue reservation.
